# Prevalence of helmintic infections in Brazilian Maxakali indigenous: a repeated cross-sectional design

**DOI:** 10.1186/s12939-024-02105-7

**Published:** 2024-02-01

**Authors:** Maria Beatriz Pena e Silva Leite Nacife, Liliane Maria Vidal Siqueira, Keila Furbino Barbosa, Valeska Natiely Vianna, Cássio Zumerle Masioli, Jaime Costa da Silva, Fábio Zicker, Paulo Marcos Zech Coelho, Naftale Katz, George Luiz Lins Machado-Coelho

**Affiliations:** 1 Universidade Federal de Ouro Preto, Escola de Medicina, Laboratório de Epidemiologia, Rua Dois s/n, sala 203, Campus Universitário Morro do Cruzeiro, Ouro Preto, Minas Gerais CEP 35402-145 Brasil; 2 Fundação Oswaldo Cruz, Instituto René Rachou, Belo Horizonte, Minas Gerais Brasil; 3 Ministério da Saúde, Distrito Sanitário Especial Indígena/Minas Gerais-Espírito Santo, Belo Horizonte, Minas Gerais Brasil; 4 Fundação Oswaldo Cruz, Rio de Janeiro, Brazil

**Keywords:** Schistosomiasis, Soil-transmitted helminths, Maxakali indigenous, Neglected Tropical Diseases

## Abstract

**Background:**

The prevalence of intestinal parasites is known to be high among Amerindian populations; further, there are serious problems in the healthcare of these populations in Brazil. The Maxakali, located in the northeastern region of Minas Gerais, Brazil, is an indigenous group that still preserves many of its cultural aspects. This study aimed to compare the positivity rate of schistosomiasis and soil-transmitted helminths in this ethnic group in epidemiological surveys conducted in 1972 and 2014.

**Methods:**

Stool parasitological examinations were performed by the Kato-Katz technique during both periods in this population. In 2014, the parasitological diagnosis was also realized with the TF-Test® technique.

**Results:**

In 1972, 270 inhabitants were examined. The positivity rates were 67.4% for *Schistosoma mansoni*, 72.9% for hookworms, 43.7% for *Ascaris lumbricoides*, and 23.7% for *Trichuris trichiura*. In 2014, 545 individuals were examined, and the positivity rates obtained were 45.7% for *S. mansoni*, 22.8% for hookworms, 0.6% for *A. lumbricoides,* and 2.8% for *T. trichiura*.

**Conclusions:**

The comparison of the parasitological surveys conducted in 1972 and 2014, indicates that the indigenous Maxakali remained neglected by the health and indigenous protection authorities during these four decades. The infection rate observed in 2014 for schistosomiasis and hookworm remains high, considering the current epidemiological view of these diseases in the Brazilian population.

## Background

Infectious and parasitic diseases, often called neglected tropical diseases (NTDs) and caused by parasites and infectious agents, mainly affect socioeconomically disadvantaged populations, such as poor populations in Africa, Asia, and Latin America [1–5]. The main NTDs include soil-transmitted helminths, schistosomiasis, leishmaniasis, lymphatic filariasis, rabies, African trypanosomiasis, Chagas' disease, cysticercosis, onchocerciasis, trachoma, and echinococcosis [6].

The highest prevalence of these diseases is in the tropical regions with the world's poorest populations; these diseases can lead to premature death, suffering, and disability in the long term [3]. It is estimated that millions of people are affected by these diseases; however, information on morbidity and disability related to them remains scarce [7].

NTDs usually affect neglected populations in developing countries, where some individuals are more susceptible to parasitic infections and/or other transmissible diseases. These individuals include indigenous populations, ethnic minority communities, children, the elderly, and immunocompromised individuals [8].

Diseases caused by intestinal parasites rank high among major infections affecting the world's population. They are caused by helminths and protozoa, which lodge in different parts of the host's intestine, and may also impact other organs, such as the liver and lungs [9]. These diseases result in high morbidity rates and serious health problems that can lead to learning and cognitive disorders [10]. Intestinal parasitic infections are considered neglected diseases because they are not given due importance by government agencies [11, 12]. To prevent these diseases, it is imperative to improve socioeconomic conditions and hygiene; inculcate positive cultural habits; and implement basic sanitation and health education [13].

Intestinal parasites are present throughout Brazil, mainly affecting children and socioeconomically disadvantaged communities; they continue to remain a serious public health problem in the country [14]. Whereas the Brazilian health quality has improved over time (e.g., increased life expectancy and fewer deaths due to infectious diseases), the health quality of the indigenous population do not show significant improvement, using these parameters [15].

Intestinal parasitic diseases in Brazilian indigenous tribes are one of the primary elements in their epidemiological profile [16]. The high prevalence of these diseases are indicative of inadequate sanitation and health infrastructure. These factors may be attributable to the Brazilian indigenous populations' contact with noindigenous locals society, which has led to a pronounced degree of social exclusion. To avoid diseases spreading over Amerindian populations, it is necessary to examine their habits, from their culture to living conditions, as well as the epidemiological profile of the diseases that affect these populations [17].

This present study was developed with the Maxakali, an indigenous group in Minas Gerais State. With a total population of approximately 2,000 inhabitants, this ethnic group is found in Água Boa, in the municipality of Santa Helena de Minas; in Pradinho, in the municipality of Bertópolis; in Aldeia Verde, in the municipality of Ladainha; and in Aldeia Cachoerinha, Topázio District, municipality of Teófilo Otoni, in the northeastern region of Minas Gerais state [18].

The Maxakali group is marked by a history of high mortality rates, caused by malnutrition, alcoholism, homicides arising from internal conflicts, diarrheal diseases, scabies, and respiratory diseases [19]. Amerindians of this ethnic group do not wash their hands and food before meals. They walk barefoot and cook over makeshift fires on a ground outside the house, using a single pot. Additionally, they deal with the presence of a large amount of feces around their dwellings, all of which engender the presence of parasitic diseases. Intestinal parasite infection is further exacerbated by the fact that they live in agglomerates, in houses with earthen floors, have few toilets, generally unused, and drink untreated, poor-quality water stored in buckets and pans [19].

The present study aimed to compare the prevalence of schistosomiasis mansoni and soil-transmitted helminths in the Maxakali group, through epidemiological surveys conducted in 1972 and 2014.

## Methods

### Area and period of the study

This study was carried out in two distinct periods, 1972 and 2014, in two groups of Maxakali living around Água Boa and Pradinho health centers. In 1972, the Maxakali population comprised 277 inhabitants; in 2014, this number increased to 1,546.

### Stool examination

Parasitological diagnosis in the 1972 and 2014 surveys was carried out by using the Kato-Katz (KK) quantitative technique [20]. In the last survey, the Helmtest® kit (Biomanguinhos, FIOCRUZ, Rio de Janeiro, RJ, Brazil) was used, according to the manufacturer's protocol. In 2014, one slide of each stool sample was examined, corresponding to 41.7 mg of feces. The quantitative exam was performed for *S. mansoni* and the number of eggs per gram of feces (epg) was calculated by multiplying the number of eggs detected in each slide by the correction factor 24.

In 2014, the parasitological diagnosis was also made with the TF-Test®, a commercial technique that was also carried out under the manufacturer's recommendations (Bio-Brasil Biotecnologia, Anápolis, GO, Brazil); however, only one fecal sample was used. In short, a small portion of feces was added in a 10% formalin-containing tube, in which 3 mL of ethyl acetate and one drop of detergent were also added, and then centrifuged at 500 g for 1 min 30 s. The sediment generated during centrifugation was examined under an optical microscope in 10 × and 40 × increments to identify helminths (larvae and eggs) and protozoa (cysts), with three slides of each sample being analyzed.

### Parasitological survey

In 2014, sterile specimen cups were provided to this population (*n* = 1546), of which 616 samples were returned, achieving a 39.8% adherence rate. The subjects in the study included those living in the above-mentioned villages, of both sexes, regardless of age, and those who agreed to participate in the study. Those who did not complete the two diagnostic tests were excluded. In the end, a total of 545 samples were analyzed: 296 (54.3%) females and 249 (45.7%) males.

### Statistical analysis

The program GraphPad Prism version 5 built the graphical figures. Differences between proportions were tested by the Qui-square test of Pearson using the Epi Info 7.2.0.1. The significance level was defined by the p-value (*p* < 0.05).

## Results

**Fig. 1:**
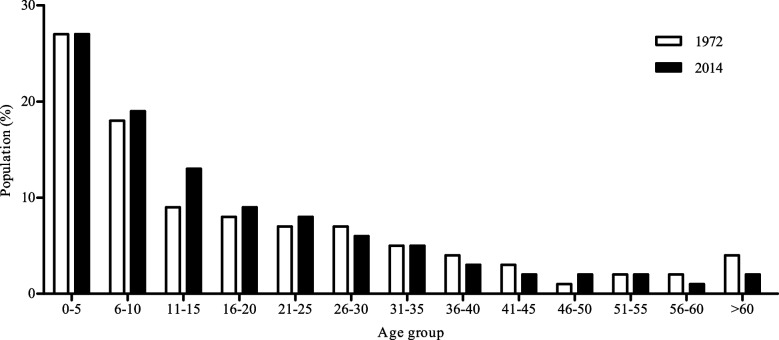
Demographic profile of the population study stratified by age group, in the years 1972 and 2014

The demographic profile of the Maxakali indigenous population on the two epidemiological surveys is shown in Fig. 1. The distribution of individuals according to age demonstrates that the Maxakali group comprises mostly of early age population (0–10 years).

**Fig. 2:**
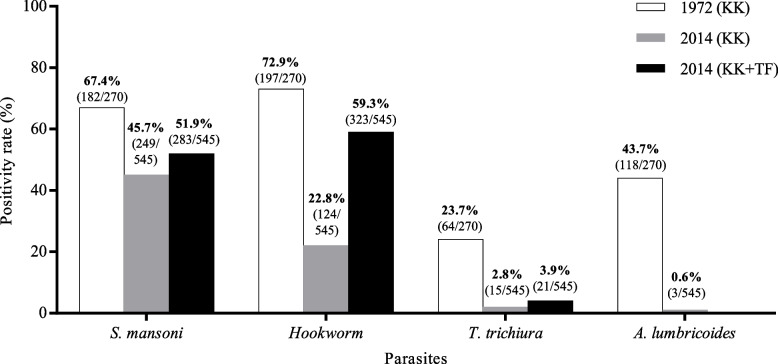
Positivity rates of helminths, in the surveys of 1972 and 2014, in the Maxakali indigenous ethnic group, Brazil

Figure 2 shows the positivity for helminths in both surveys. In the survey conducted in 1972, 270 subjects participated in the study, of which 67.4% were positive for *S. mansoni*, 72.9% for hookworm, 43.7% for *A. lumbricoides,* and 23.7% for *T. trichiura*. In 2014, the positivity rates for these parasites were 45.7%, 22.8%, 0.6% and 2.8%, respectively. Considering the Kato-Katz and TF-Test® combination (used only in the 2014 survey), results showed that of the 545 participants, 459 tested positive for at least one helminth (84.2%). The positivity for *S. mansoni* increased from 45.7% to 51.9%; for hookworm, from 22.8% to 59.3%; and for *T. trichiura*, from 2.8% to 3.9%. Positivity for *S. mansoni* using only the Kato-Katz technique was 45.7% and for only the TF-Test® technique, 33.2%. (*p* < 0.05). For hookworms, the highest rate was obtained using the TF-Test®, with a positivity of 46.8%, and with the Kato-Katz, the positivity was 22.8%. (*p* < 0.05). *A. lumbricoides* eggs (0.6%) were detected only by the Kato-Katz technique.

**Fig. 3:**
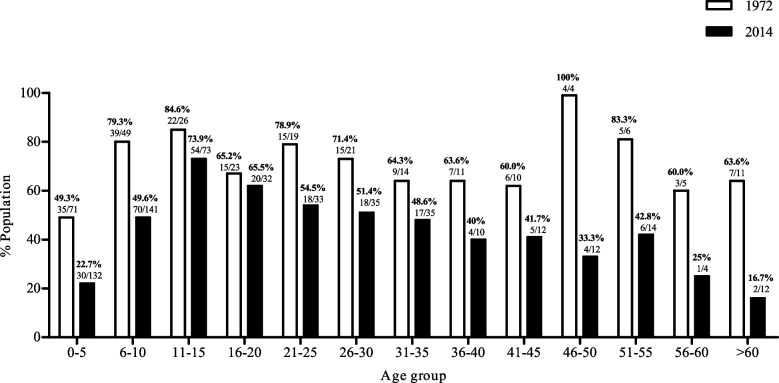
Positivity rate of *S. mansoni* obtained by the Kato-Katz technique, stratified by age group in the surveys of 1972 and 2014, in the Maxakali ethnic group

Figure 3 shows the positivity for *S. mansoni* according to the Kato-Katz technique by age group in both surveys. The results demonstrate that regardless of the age group, the positivity rates were higher in 1972 than in 2014, with greater differences in the age group from 0–10, and from 45– 60 and above. In the 1972 survey, the highest rates were observed in the 46–50 age group, followed by the 6–15 age group. Conversely, in the 2014 survey, the highest positivity rates were obtained for the 11–15 age group, decreasing with an increase in age. The peak in positivity in the 46–50 (100%) age group, observed in the 1972 survey, must be analyzed cautiously due to the low number of participants in these age groups.

## Discussion

The prevalence of intestinal parasites is one of the best indicators of a population's socioeconomic profile [21]. Although there have been notable improvements in the quality of life in Brazil's population in the last decades, the prevalence of intestinal parasites in indigenous communities continues to remain high. This may be because the environment in which they live favors the development of such parasites. Such an environment includes heat and humidity, along with an absence of water and sewage treatment, as well as precarious socioeconomic conditions [22].

The indigenous Maxakali area, in the Mucuri valley, in the Northeastern region of Minas Gerais state, has environmental characteristics that engender the dissemination of parasites and the development of larval forms of soil-transmitted helminths [19, 23]. It is also a plain region, with a lentic hydrographic basin and vertical or floating vegetation,this propitiates the development of the genus *Biomphalaria* sp, intermediate hosts of *S. mansoni* [24] A recent malacological survey found *Biomphalaria* snails in 79 out of 120 municipalities in Minas Gerais State [25]. The species *B. glabrata* and *B. straminea* were found in the municipalities of Santa Helena de Minas and Bertópolis, respectively (where the Maxakali indigenous villages are located). However, the snails were not found infected with *S. mansoni*. It is worth noting that the positivity of the intermediate hosts in endemic areas are varied due to diagnostic techniques, the state of biological preservation, the time of examination, and the species of the snail [26]. However, the hosts’ presence indicates a potential endemic area.

The high prevalence of intestinal parasitoses in indigenous lands occurs because they suffer social exclusion and sedentarization. Indigenous peoples live in restricted areas without basic sanitation, eat with unwashed hands, defecate in the soil, walk barefoot, bathe in natural waters (rivers and lakes), and do not have ways to conserve food, which facilitates infection by intestinal parasites [27].

According to Assis et al. [19], the Maxakali people have preserved their language and cultural habits, that facilitates parasite transmission. They live in houses made of mud, unfinished or covered with canvas, and have few toilets that are seldom used (only during village festivities). The villages are organized in familiar unit houses, with no water or sewage treatment,further, water is stored in unsuitable places [19].

According to information provided by the Special Indigenous Sanitary District (DSEI—MG/ES), considering the semi-nomadism of this population in the 2014–2019 period, the rate of latrines per household in the Água Boa and Pradinho health centers was 25% (30/119) and 4.6% (6/130) respectively, with no significant variation in the period. These data indicate a low level of sanitation and sewage infrastructure in the Maxakali indigenous land.

The Amerindian populations are among the most neglected, with a greater risk of social, economic, and cultural exclusion [19]. Comparing the prevalence of helminths in the two epidemiological surveys (1972 and 2014) conducted with the Maxakali population in a gap of four decades, a decrease in the rates of positivity of helminths was observed,however, these rates remain high, from 67.4% to 45.7% for *S. mansoni*, and from 72.9% to 22.8% for hookworms. A sharp decrease was found regarding *Ascaris* and *Trichuris*, decreasing from 43.7% and 23.7% to 0.6% and 2.8%, respectively. This reduction may have been due to repeated anti-helminthic treatments carried out over years, which are safe, with low toxicity, low cost, and a single oral dose [28].

In 1972, the treatment for soil-transmitted helminths was conducted three times, with a six-month interval between each. Schistosomiasis treatment was administered annually to people with *S. mansoni* eggs in their feces, for two consecutive years. From 1975, one treatment was done by local health services, based on passive demand. In 2014, mass treatment was realized by health service, which may have contributed to the reduction of parasitic prevalence [19, 29].

The decrease in the prevalence of *T. trichiura* (from 23.7% to 2.8%) and *A. lumbricoides* (from 43.7% to 0.6%) may confirm the efficacy of chemotherapy, by using broad-spectrum antihelminthic, such as mebendazole and albendazole that are routinely used in Brazilian health services. In 1972, the treatment for soil-transmitted helminths was made three times, with a six-month interval between each. From 1975, the treatment was done by local health services.

Based on a study conducted on the prevalence of *A. lumbricoides* in an urban area of São Paulo (SP), Ferreira et al. [30] stated, that areas with high demographic density, without adequate housing and sanitation, have a higher risk of infection than rural areas. In indigenous populations, the seminomandism of the inhabitants can explain the low prevalence of *A. lumbricoides* and *T. trichiura* observed in the present study. It is important to emphasize that no anthelminthic treatment was carried out in the study area with a defined periodicity in the most recent years, which justifies the high prevalence of the other helminths.

The risk of *T. trichiura* infection, despite being mainly environmentally influenced, also significantly involves genetic and household components, which may explain its low prevalence [31, 32].

Regarding the reduction in the positivity of hookworms from 72.9% in 1972 to 22.8% in 2014, both obtained by the Kato-Katz technique, the rates may be underestimated. The hookworms’ egg structure starts to fade and eventually becomes invisible after a six-hour preparation in the slide [28]. Considering the combination of both techniques (Kato-Katz and TF-Test®) used in the last survey, the positivity rate for hookworms increased to 59.3%, demonstrating that the infection by this parasite remained high in this ethnic group. The greater detection of hookworms by the TF-Test® may be due to the biological conservation of the samples with formaldehyde.

Soil characteristics in the Maxakali villages are also favorable for the development of the larval stages of soil-transmitted helminths [19, 23], which explains the prevalence of hookworm. Studies in other indigenous villages in Brazil also demonstrated the presence of these helminths. Coimbra-Jr and Mello [33]  obtained a prevalence of 43.3% for hookworms in the indigenous people of Suruí of the Aripuanã Park, in Rondônia state. Miranda et al. [34], in a study conducted in the Parakanã indigenous community in the Pará state, found a prevalence of 33.3%.

*S. mansoni* also continues to present a high positivity rate. In 1972, the rate was 67.4%, which fell to 45.7% in 2014; both rates were obtained by the Kato-Katz technique. Despite being significant, the positivity rate remains high. The fact that the Maxakali are semi-nomadic [19], the presence of intermediate hosts in the region, frequent contact with potentially contaminated natural waters, cultural habits, and lack of basic sanitation, contribute to the persistence of schistosomiasis among this indigenous population [28, 35]. The low acceptance of treatment by the population further exacerbates the prevalence of schistosomiasis, explaining the higher positivity rate.

The Maxakali’s indigenous behavior of taking collective baths in natural water may further facilitate schistosomiasis infection. This is because this population shares the same water for different chores (baths, washing, cleaning, fishing); and because they use a characteristic instrument (called *"puçá"*), for fishing, which requires them to enter the aquatic environments [19].

In Brazil, three national surveys have already been conducted to assess the prevalence of schistosomiasis mansoni and two for soil-transmitted helminths. The first, between 1949 and 1953, was coordinated by Pellon and Teixeira [36],the second (1975–1979), by the Special Program for Schistosomiasis Control – PECE – [24] ; and the third (2010/2015) called the National Survey on Prevalence of Schistosomiasis mansoni and soil-transmitted helminths (INPEG) by Katz [28]. In this last survey, it was possible to observe a sharp decrease in the prevalence of schistosomiasis and soil-transmitted helminths. In 11 endemic states for schistosomiasis, the positivity decreased from 10.09% in the first survey to 9.24% in the second and 1.79% in the most recent one. The PECE survey showed that in the region where the Maxakali are located, the prevalence was 14.5% for schistosomiasis. Regarding soil-transmitted helminths, in the Minas Gerais state, the positivity for these infections was 89.4% in the first survey, whereas in the last one, the positivity was 1.4% for ascariasis, 0.9% for hookworm, and 0.6% for trichuriasis.

These sharp reductions in the positivity rates of schistosomiasis and hookworm were not found in the indigenous Maxakali; however, the decrease of the prevalence of ascaridiasis and trichuriasis was similar to what has been observed in the state, indicating that this population needs greater attention, access to health, and better control strategies. Several factors, such as improvement in the sanitation conditions in the regions of the country and the specific treatment carried out in endemic areas, may have contributed to the decrease in the positivity rates of these parasite infections and the morbidity and mortality of schistosomiasis in Brazil [28].

The latest National Survey of Basic Sanitation (PNSB) conducted by the Brazilian Institute of Geography and Statistics in 2010 [37] , in agreement with the Cities Ministry in 2008, showed that in Minas Gerais state, all of its 853 municipalities presented some type of basic sanitation service (general water distribution network, sewage collection system, solid waste management and/or stormwater management). Considering the state water distribution network, all municipalities had access to this service; however, 8.1% did not receive treated water. Regarding the sewage collection system, 91.6% of the municipalities of the state had sewage collection systems, but only 22.7% treated the sewage. Nevertheless, this is not the reality on Maxakali indigenous lands, since the environmental and cultural aspects, inherent to this population, make it difficult to implement these sanitation actions. Moreover, they are neglected by the governmental authorities.

While there are significant findings, this study had some limitations, including the use of a single fecal sample per individual for the execution of the Kato-Katz (one slide) and TF-Test® techniques, which might have entailed a loss of sensitivity. This methodological decision was made to avoid logistic problems to carry out and conduct a study and due to the difficulty in working in the indigenous area. This limitation was partly compensated by the association of the TF-Test® with the Kato-Katz technique.

## Conclusions

The data from this study indicate that infections by schistosomiasis and hookworm are still high, reflecting an urgent need for intervention by implementing prevention measures (health education) and control strategies, such as large-scale treatment associated with improved sanitary conditions through water supply, basic sanitation, and not walking barefoot. A comparison of the results of parasitological surveys indicates that the indigenous Maxakali ethnicity has remained neglected over the decades. Health and environmental authorities must act immediately to implement control measures aiming to improve the health situation of this population.

## Data Availability

Not applicable.

## Abbreviations

*S. mansoni*: *Schistosoma mansoni*
*A. lumbricoides*: *Ascaris lumbricoides*
*T. trichiura*: *Trichuris trichiura*
NTDs: Neglected tropical diseases
KK: Kato-Katz quantitative technique
RJ: Rio de Janeiro
Epg: Eggs per gram of feces
FUNAI: National Indian Foundation
*B. straminea*: *Biomphalaria straminea*
DSEI: Special Indigenous Sanitary District
MG: Minas Gerais
ES: Espírito Santo
SP: São Paulo
PECE: Special Program for Schistosomiasis Control
INPEG: National Survey on Prevalence of Schistosomiasis mansoni and soil-transmitted helminths
PNSB: National Survey of Basic Sanitation
